# Supplementary material for: Analysis of the spatio-temporal coupling coordination mechanism supporting economic resilience and high-quality economic development in the urban agglomeration in the middle reaches of the Yangtze River
Source: PLoS One. 2023 Feb 24;18(2):e0281643. doi: 10.1371/journal.pone.0281643 (PMC9955981; doi:10.1371/journal.pone.0281643)
Supplement: Attachment — Submitted filename: Comments.docx [file pone.0281643.s001.docx]

The paper titled "Research on the spatial-temporal coupling mechanism of economic resilience and high-quality economic development in the urban agglomeration in the middle reaches of the Yangtze River" . Overall, the paper's quality can be improved; however, the following major revisions can be considered:

(1) Abstract needs to be rewritten well. It is necessary to highlight the study's main findings without mentioning that the policy and limits are suggested at the end of the article. Elaborate abstract in more detail in terms of research methodology, including data duration, and specific policy implication.

(2) Figure 1 and Figure 3 are blurred and indistinct. It is suggested to make the drawing again.

(3) Many punctuation marks do not have Spaces between words. Please use proper punctuation.

(4) The language needs further polishing, and it is best to invite a native English speaker of relevant academic research to help with the revision.

(5) I recommend authors to explicitly specify the novelty of this research paper?

(6) The introduction needs to be expanded further to cover all the critical aspects of the topic. I suggest authors to consider the following articles for citations, it will strengthen the quality of this draft:

doi.org/10.3390/su14074212

doi:10.3390/ijerph16173076

doi.org/10.3390/ijerph18115697

doi.org/10.3390/ijerph18179105

doi.org/10.3390/ijerph19020958
